# Serum insulin-like growth factor-1 and its binding protein-7: potential novel biomarkers for heart failure with preserved ejection fraction

**DOI:** 10.1186/s12872-016-0376-2

**Published:** 2016-10-21

**Authors:** Michael Coll Barroso, Frank Kramer, Stephen J. Greene, Daniel Scheyer, Till Köhler, Martin Karoff, Melchior Seyfarth, Mihai Gheorghiade, Wilfried Dinh

**Affiliations:** 1 Klinik Königsfeld der Deutschen Rentenversicherung Westfalen in Ennepetal (NRW), University Hospital, Witten/Herdecke, Germany; 2 Drug Discovery, Clinical Sciences - Experimental Medicine, Bayer Pharma AG, Leverkusen, Germany; 3 Division of Cardiology, Duke University Medical Center, Durham, NC USA; 4 Department of Cardiology, HELIOS Clinic Wuppertal, University Hospital Witten/Herdecke, Wuppertal, Germany; 5 Center for Cardiovascular Innovation, Northwestern University Feinberg School of Medicine, Chicago, USA

**Keywords:** Heart failure, Preserved, IGF-1, IGFBP-7

## Abstract

**Background:**

Insulin-like growth factor binding protein-7 (IGFBP-7) modulates the biological activities of insulin-like growth factor-1 (IGF-1). Previous studies demonstrated the prognostic value of IGFBP-7 and IGF-1 among patients with systolic heart failure (HF). This study aimed to evaluate the IGF1/IGFBP-7 axis in HF patients with preserved ejection fraction (HFpEF).

**Methods:**

Serum IGF-1 and IGFBP-7 levels were measured in 300 eligible consecutive patients who underwent comprehensive cardiac assessment. Patients were categorized into 3 groups including controls with normal diastolic function (*n* = 55), asymptomatic left ventricular diastolic dysfunction (LVDD, *n* = 168) and HFpEF (*n* = 77).

**Results:**

IGFBP-7 serum levels showed a significant graded increase from controls to LVDD to HFpEF (median 50.30 [43.1-55.3] vs. 54.40 [48.15-63.40] vs. 61.9 [51.6-69.7], respectively, *P* < 0.001), whereas IGF-1 levels showed a graded decline from controls to LVDD to HFpEF (120.0 [100.8-144.0] vs. 112.3 [88.8-137.1] vs. 99.5 [72.2-124.4], *p* < 0.001). The IGFBP-7/IGF-1 ratio increased from controls to LVDD to HFpEF (0.43 [0.33-0.56] vs. 0.48 [0.38-0.66] vs. 0.68 [0.55-0.88], *p* < 0.001). Patents with IGFB-7/IGF1 ratios above the median demonstrated significantly higher left atrial volume index, E/E’ ratio, and NT-proBNP levels (all *P* ≤ 0.02).

**Conclusion:**

In conclusion, this hypothesis-generating pilot study suggests the IGFBP-7/IGF-1 axis correlates with diastolic function and may serve as a novel biomarker in patients with HFpEF. A rise in IGFBP-7 or the IGFBP-7/IGF-1 ratio may reflect worsening diastolic function, adverse cardiac remodeling, and metabolic derangement.

## Background

Although advances in drug and device-based therapies have substantially improved survival for patients with chronic heart failure (HF) with reduced ejection fraction (HFrEF), there has been no such parallel progress with therapy for HF with preserved ejection fraction (HFpEF) [1]. The underlying reasons for the failure to identify effective treatments are incompletely understood, but major challenges relate to accurate diagnosis and the heterogeneity of the broad HFpEF patient population [2]. In this context, new and emerging biomarkers may be helpful to better define distinct pathophysiology and guide targeted therapeutic strategies. The growth hormone/insulin-like growth factor-1 (GH/IGF-1) system is essential in the regulation of growth and cellular differentiation in various tissues, with IGF-1 as the primary mediator. IGF-binding protein 7 (IGFB-7) binds to IGF-1 and neutralizes its activity, thus the IGFBP-7/IGF-1 ratio may serve as a proxy for GH system activity [3, 4]. Among HFrEF patients, activation of the GH/IGF-1 system has demonstrated prognostic significance, with levels of both serum IGF-1 and IFGBP-7 predicting clinical outcomes [3–8]. However, the potential role of these markers in among patients with preserved ejection fraction (EF) is unclear. The aim of the present study was to investigate the association between IGF-1 activity, represented by the ratio of serum IGF-1 to IGFBP-7 concentrations, with severity of echocardiographic left ventricular (LV) diastolic dysfunction in a well-phenotyped cohort of subjects with normal EF.

## Methods

This study enrolled consecutive patients referred for elective coronary angiography and echocardiography. Exclusion criteria included left ventricular EF < 50 %, the combination of grade 1 diastolic dysfunction with symptoms suggestive of HF, and the need for coronary revascularization with either angioplasty or coronary bypass surgery. Additional exclusion criteria included myocardial infarction <6 weeks prior, hypertrophic cardiomyopathy, moderate-to-severe valvular heart disease, uncontrolled hypertension, uncontrolled atrial fibrillation or other severe arrhythmias, or serum-creatinine > 2.0 mg/dl. The study was approved by the local ethics committee of the University of Witten/Herdecke and was conducted in accordance with the Declaration of Helsinki. Signed informed consent was obtained from all patients.

Echocardiography was performed using a standard ultrasound system (Vivid 7, General Electric, Milwaukee, Wisconsin). Left ventricular EF was measured based on the modified biplane Simpson’s method. The left atrium volume index (LAVi) was calculated using the biplane area-length method. Dimensions were recorded by standard techniques according to current guidelines [9]. Left ventricular mass index (LVMi) was calculated by the Devereux formula indexed to the body surface area. Conventional transmitral flow was measured with pulse wave Doppler. Early (E) and late atrial (A) transmitral peak flow velocities and the ratio (E/A) were measured and three consecutive beats were averaged. Pulsed wave tissue Doppler imaging (TDI) was performed at the junction of the septal and lateral mitral annulus and three consecutive beats were averaged. Early diastolic velocities (E’ medial, E’ lateral) were recorded; the mean value (E’ average) of E’ at the medial and lateral mitral annulus was determined. Ratios of E/E’ medial, E/E’ lateral and average E/E’ ratio were calculated. Diastolic dysfunction was classified according to the consensus study by the American and European Society of Cardiology [10]. Patients were categorized into 1 of 3 groups: controls, asymptomatic left ventricular diastolic dysfunction (LVDD), and HFpEF. Controls were defined by diastolic function (E/E’ < 8 and normal left atrial volume index). LVDD was defined as grade I diastolic dysfunction without clinical HF symptoms of HF. HFpEF was defined as LVDD grade II or III with or without clinical symptoms or signs of HF. Signs and symptoms HF included, but not limited to fluid retention (e.g. ankle swelling), shortness of breast, reduced exercise tolerance and fatigue [11]. As mentioned, patients with grade I diastolic dysfunction with symptoms suggestive of HF were excluded.

Blood samples were drawn at rest for the analysis of routine laboratory parameters. The blood tubes were centrifuged at 2000 g at room temperature for 10 min, and serum or plasma were separated from cellular compartments and stored at −80 °C for later analysis of IGF-1, IGFBP-7, N-terminal pro-B-type natriuretic peptide (NT-proBNP) and soluble suppression of tumorigenicity-2 (sST2). After thawing, plasma concentrations of IGF-1 and sST2 were measured by enzyme-linked immunosorbent assays (ELISA) in accordance with the manufacturer’s instructions (human IGF-I Quantikine ELISA Kit, human ST2/IL-1 R4 Quantikine ELISA Kit, R&D Systems, Minneapolis, MN, USA). IGFBP-7 was measured using a novel sandwich immunoassay that was developed and validated using a microtiter plate prototype ELISA (Roche Diagnostics, Penzberg, Germany). The limit of detection for the IGFBP-7 assay was 0.10 ng/mL. The inter-run and intra-run coefficients of variation were 4.8 % and 3.5 % at concentrations of 38.0 ng/mL and 26.0 ng/mL. Levels of NT-proBNP were measured with the electrochemiluminescence (ECLIA) immunoassay for NT-proBNP (Roche Diagnostics, Germany) [12]. In patients without diabetes, a standardized oral glucose tolerance test (oGTT) was performed according to the World Health Organization protocol as previously described [13]. Metabolic syndrome was diagnosed according to the amended National Cholesterol Education Program’s Adult Treatment Panel III (ATP-III) guidelines [14].

Baseline characteristics for controls, LVDD patients, and HFpEF patients were compared. IGFBP-7/IGF-1 ratios were calculated for each patient. Spearman rank correlation was used to test association between between IGFBP-7/IGF-1 ratio and age, NT-proBNP, and sST2 and between log IGF axis and body mass index (BMI), waist circumference, hip circumference, glucose levels and the homeostasis model assessment of insulin resistance (HOMA) index. IGF-1, IGFBP-7 and IGFBP-7/IGF-1 axis levels were compared across the 3 patient groups and different categories of the E/E’ average ratios and the left atrial volume index by the Jonckheere-Terpstra test. A multivariable model was included to predict presence of HFpEF and included the following covariates: age, gender, BMI, coronary artery disease, hypertension, NT-proBNP, and IGFBP-7/IGF-1 ratio.

Continuous variables were reported as medians (interquartile range) and categorical variables were reported as frequencies and percentages, unless otherwise specified.

Log-transformed values were used as appropriate. Non-parametric tests for group differences between categories were performed. We used the Kruskal-Wallis or Jonckheere-Terpstra test to test the equality of medians among more than two distinct groups. The Wilcoxon-Mann–Whitney *U*-test was used to analyze differences between the medians of two groups and the *χ*2 test to evaluate differences in proportions in more than 2 sets of categorical variables. Fisher’s Test was used for the comparison of two sets of binary variables. The incremental diagnostic utility of the IGFBP7/IGF-1 ratio was assessed by comparing the areas under the curve (AUCs) of receiver operating characteristics (ROC) curves. All analyses were performed using SPSS statistical software (SPSS 17.0, Chicago, IL). A *p*-value < 0.05 or less was considered statistically significant.

## Results

**Fig. 1:**
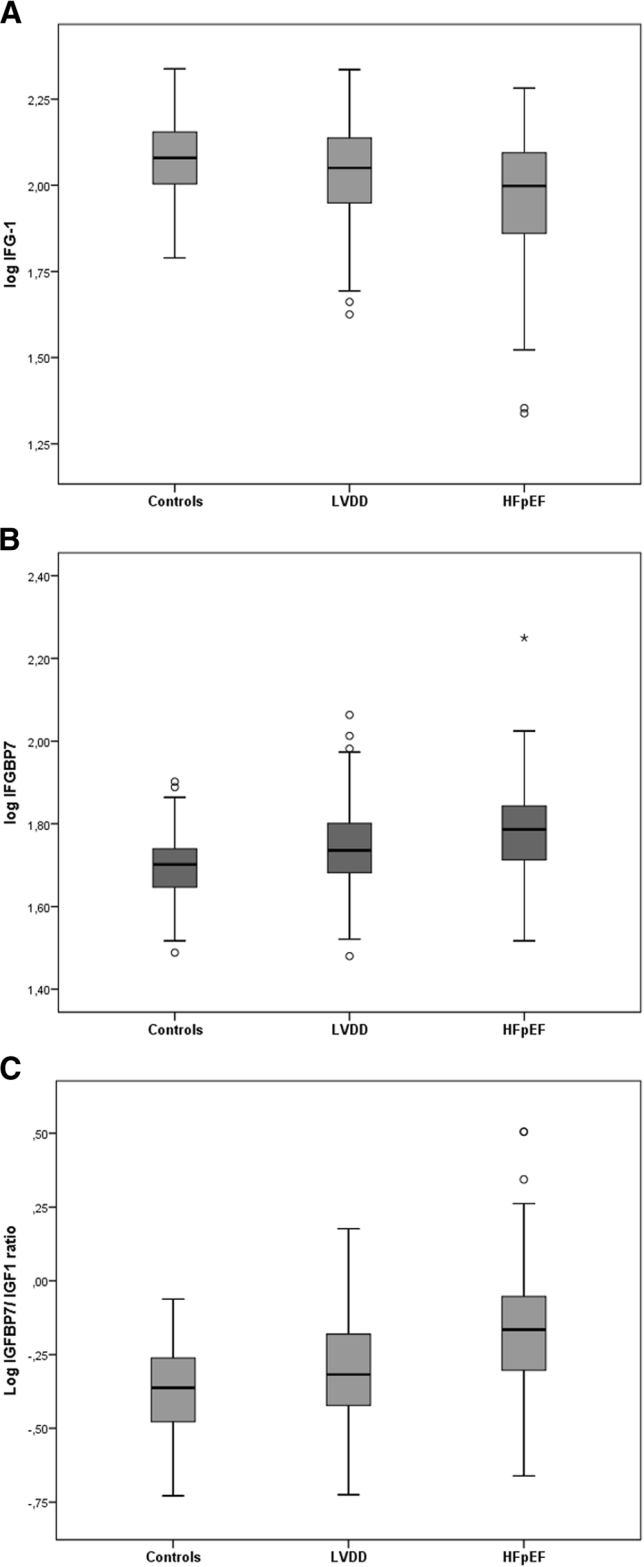
Comparison of serum insulin-like growth factor-1 (IGF-1) (**a**), and insulin-like growth factor-binding protein-7 concentrations (IGFBP-7) (**b**), and the insulin-like growth factor axis (the molar ratio of IGFBP-7 and IGF-1) (**c**) between controls, LVDD and HFpEF. Log IGFBP-7, log IGF-1 and the IGFBP7/IGF-1 levels are presented as box (25th percentile, median, 75th percentile), and whiskers plots, with outliers expressed as dots. All comparisons, *P* <0.001. LVDD, asymptomatic left ventricular diastolic dysfunction; HFpEF, heart failure with preserved ejection fraction

Overall, the study included 300 patients (mean age 64 ± 10 years; 51 % men), of which 168 patients had asymptomatic LVDD, 77 patients had HFpEF, and 55 were controls. Baseline patient characteristics are summarized in Table 1. As compared with controls, patients with LVDD and HFpEF tended to have higher BMI, weight circumference, and systolic blood pressure, and were more likely to have pre-existing hypertension, diabetes, and coronary artery disease. Levels of sST2 and NT-proBNP were highest among HFpEF patients. There was a progressive decrease in serum IGF-1 levels from controls to LVDD patients to HFpEF patients, with HFpEF patients having the lowest levels (all *P* < 0.001, respectively, Table 2, Fig. 1). In contrast, there was a progressive increase in IGFBP-7 level and IGFBP-7/IGF-1 ratio from controls to LVDD patients to HFpEF patients (all *P* < 0.001). In multivariable analysis, age (*P* =0.001), NT-proBNP (*P* < 0.001), and IGFBP-7/IGF-1 ratio (*P* =0.005) were independently associated with HFpEF.

**Table 1:** Baseline characteristics of study participants (n = 300)

Variable	Controls (*n* = 55)	LVDD (*n* = 168)	HFpEF (*n* = 77)	*p*-value
Demographics and vital signs				
Age (years)	54 [48–61]	66 [58–71]	73 [68–77]	< 0.001
Male	52.7 %	56.2 %	40.3 %	0.138
BMI (kg/m^2^)	25.5 [24.1-29.1]	27.8 [25.6- 32.3]	27.5 [25.7- 32.0]	0.008
Waist circumference (cm)	98 [86–107]	102 [94–114]	102 [98–111]	0.004
Hip circumference (cm)	98 [94–103]	103 [96–111]	105 [98–114]	0.002
Systolic BP (mmHg)	125 [110–136]	134 [127–140]	136 [130–140]	< 0.001
Diastolic BP (mmHg)	80 [70–80]	80 [76–84]	80 [72–84]	0.013*
Heart rate (bpm)	70 [68–76]	72 [69–76]	70 [65–76]	0.195
Medical history				
Hypertension	69 %	88 %	96 %	< 0.001
T2DM	12.7 %	42.6 %	53.2 %	< 0.001
CAD	38.2 %	58.6 %	63.6 %	0.023
CABG	1.8 %	3.0 %	11.7 %	0.001
PCI	5.5 %	16.1 %	13.2 %	0.155
Myocardial infarction	14.5 %	21.3 %	22.1 %	0.572
Medications				
Beta-blocker	50.9 %	60.9 %	74.0 %	0.041
ACE inhibitor	47.3 %	65.1 %	54.5 %	< 0.001
ARB	10.9 %	10.1 %	29.9 %	0.044
Diuretics	14.5 %	26.6 %	46.8 %	< 0.001
Aspirin	52.7 %	75 %	76.6 %	0.007
Calcium blockers	10.9 %	13.6 %	27.3 %	0.024
Biomarkers				
Creatinine (mg/dl)	0.8 [0.7-0.9]	0.9 [0.7-0.9]	0.90 [0.75- 1.10]	0.060
Hba1c (%)	5.7 [5.4-5.9]	6.0 [5.7- 6.6]	6.1 [5.7-6.7]	< 0.001
sST2 (ng/ml)	13.50 [9.2-20.6]	16.20 [12.35- 21.65]	16.9 [12.2- 25.9]	0.007
NT-proBNP (pg/mL)	90.10 [45.8-129.2]	86.85 [43.7-173.4]	343.6 [151.7-703.4]	< 0.001

Values are median (interquartile range) and %

*ACE* Angiotensin converting enzyme; *ARB* angiotensin II receptor blocker; *BMI* body mass index; *BP* blood pressure; *CAD* coronary artery disease; *CABG* coronary artery bypass graft; *Hba1c* hemoglobin A1c; *HFpEF* heart failure with preserved ejection fraction; *LVDD* asymptomatic left ventricular diastolic dysfunction; *NT-proBNP* N-terminal pro-B-type natriuretic peptide; *PCI* percutaneous coronary intervention; *sST2* soluble suppression of tumorigenicity-2; *T2DM* type 2 diabetes mellitus. Non-parametric tests for group differences between categories were performed. We used the Kruskal-Wallis to test the equality of medians among more than two distinct groups. The Wilcoxon-Mann–Whitney *U*-test was used to analyze differences between the medians of two groups and the *χ*2 test to evaluate differences in proportions in more than 2 sets of categorical variables

**Table 2:** IGF-1, IGFBP7 and IGFBP-7/IGF-1 ratio (*n* = 300)

Biomarker	Controls (*n* = 55)		LVDD (*n* = 168)		HFpEF (*n* = 77)	
	Median [IQ range]	Range	Median [IQ range]	Range	Median [IQ range]	Range
IGF-1 (ng/ml)	120.00 [100.80-144.00]	61.60-217.50	112.30 [88.80-137.10]	42.20-216.40	99.50 [72.20-124.40]	21.80-191.60
IGFBP-7 (ng/ml)	50.30 [43.10-55.30]	30.80-79.80	54.40 [48.15-63.40]	30.20-115.80	61.10 [51.60-69.70]	32.90-177.6
IGFBP7/IGF-1	0.43 [0.33-0.56]	0.019-0.87	0.48 [0.38-0.66]	0.19-1.50	0.68 [0.50-0.88]	0.22-3.20

*IGF-1* insulin-like growth factor-1; *IGFBP-7* insulin-like growth factor binding protein-7. We used the Kruskal-Wallis to test the equality of medians among more than two distinct groups

The diagnostic performance for the diagnosis of HFpEF was analyzed by ROC analysis for NT-proBNP, IGF-1, IGFBP7 and the IGFBP7/IGF-1 ratio in the subgroups of HFpEF patients and controls (Table 3).

**Table 3:** Receiver-operating analysis for heart failure biomarkers

Biomarker				
	AUC	Std. Error	95%CI	*p*-value
IGF-1 (ng/ml)	0.694	0.046	0.605-0.783	< 0.001
IGFBP-7 (ng/ml)	0.730	0.044	0.643-0.817	< 0.001
IGFBP7/IGF-1	0.785	0.039	0.708-0.862	< 0.001
NT-proBNP (pg/ml)	0.835	0.036	0.765-0.905	< 0.001

Diagnostic performance of biomarkers for the diagnosis of HFpEF in the subgroup of patients with HFpEF and controls. *IGF-1* insulin-like growth factor-1; *IGFBP-7* insulin-like growth factor binding protein-7. *CI* confidence interval. T-proBNP, N-terminal pro-B natriuretic peptide

**Fig. 2:**
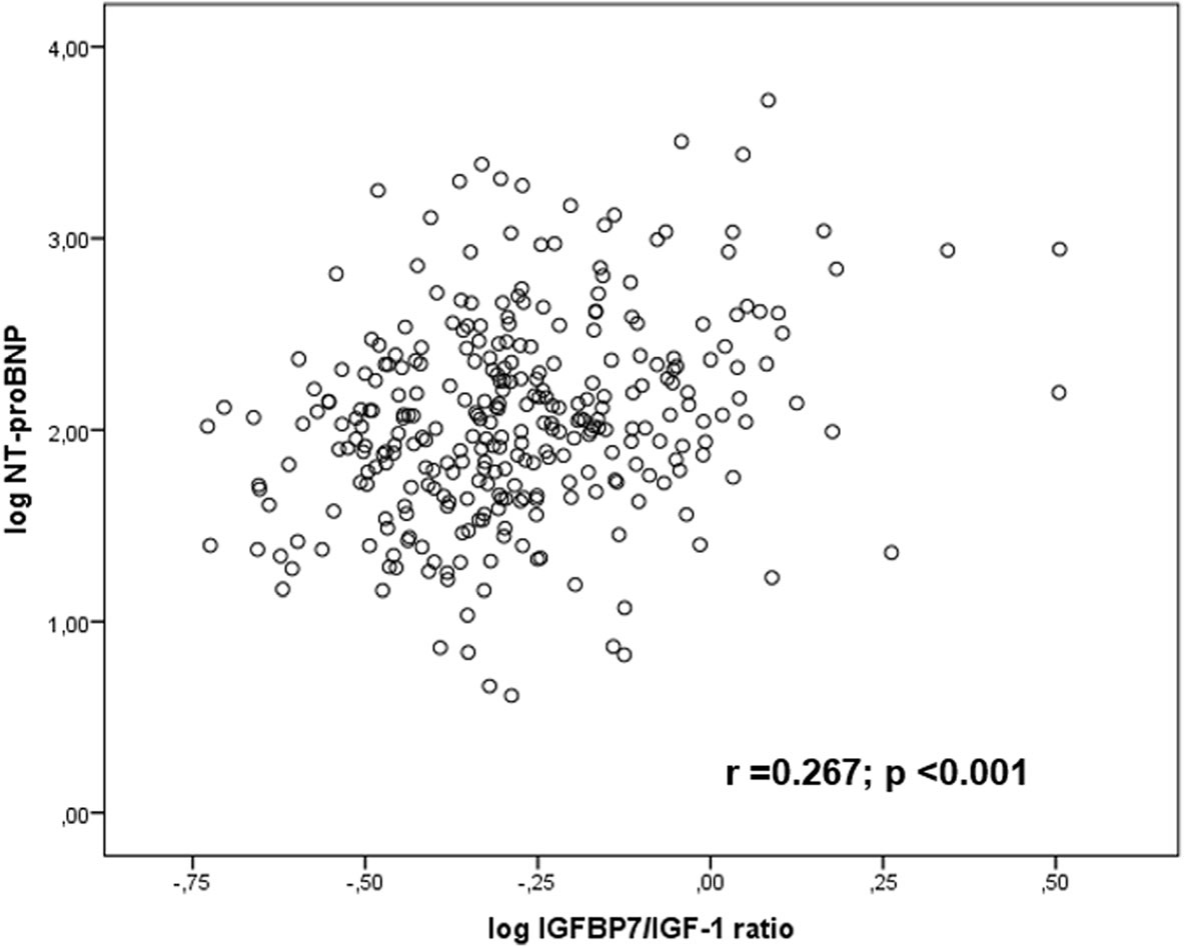
Correlation between log IGFBP-7/IGF-1 ratio and log NT-proBNP. NT-proBNP, N-terminal pro-B-type natriuretic peptide

**Fig. 3:**
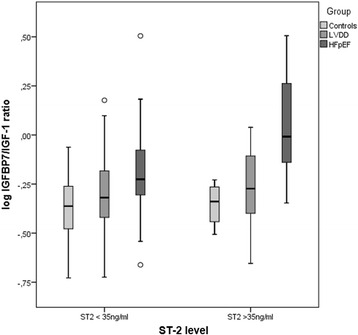
IGFBP-7/IGF-1 ratio between controls, LVDD, and HFpEF, stratified by soluble ST2 level < or ≥ 35 ng/mL. LVDD, asymptomatic left ventricular diastolic dysfunction; HFpEF, heart failure with preserved ejection fraction

**Fig. 4:**
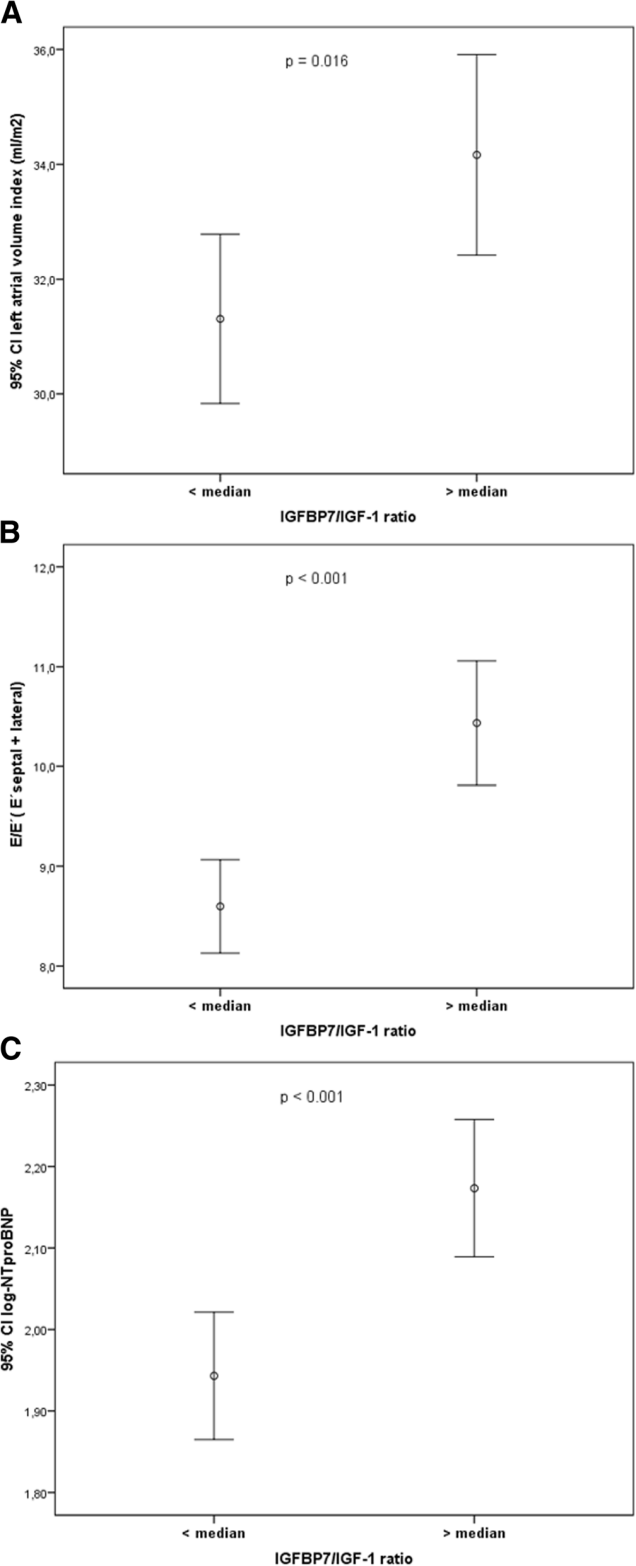
Comparison of left atrial volume index (**a**), average E/E’ ratio (septal and lateral averaged) (**b**), and NT-proBNP levels (**c**) for patients above and below the median IGFBP-7/IGF-1 ratio

IGFBP-7/IGF-1 ratio was positively correlated with age (*r* =0.471; *p* < 0.001) and other prognostic biomarkers including NT-proBNP (*r* =0.267, *p* <0.001, Fig. 2), sST2 (*r* =0.157, *r* =0.006) and hs-CRP (*r* =0.182, *p* =0.002). When the study population was stratified by sST2 level, HFpEF patients with sST2 ≥ 35 ng/ml had higher IGFBP-7/IGF-1 ratios than HFpEF patients with < 35 ng/ml (*P* =0.022, Fig. 3). Patents with IGFB-7/IGF-1 ratios above the median demonstrated significantly higher LAVi, E/e’ ratio, and NT-proBNP levels compared to patients below the median (all *P* ≤ 0.02, Fig. 4). The left ventricular mass index (LVMi) and global longitudinal strain (GLS) was significantly different between the study group (all *p* < 0.05), however, there were no correlation between median IGFBP-7/IGF-1 ratio and the or GLS (all *P* > 0.05, respectively).

In assessment of associations between IGFBP-7/IGF-1 and metabolic function, significant positive correlations were found with BMI (*r* =9.193, *P* =0.001), waist circumference (*r* =0.184, *p* =0.002) and hip circumference (*r* =0.244, *P* < 0.001). In the overall study populations, patients with metabolic syndrome showed significant increase in the IGFBP-7/IGF-1 ratio compared to patients without metabolic syndrome (0.53 [0.41-0.74] vs. 0.47 [0.36-0.59], respectively, *p* =0.002). Similarly, the IGFBP-7/IGF-1 ratio was higher in subjects with diabetes compared to patients without diabetes (0.56 [0.43-0.79] vs. 0.47 [0.36-0.60], respectively, *P* < 0.001)

## Discussion

To our knowledge, this is the first published report linking the IGFBP-7/IGF-1 axis to the presence and severity of diastolic function abnormalities and HFpEF, thus identifying a potential new candidate biomarker for this population. In the present study, higher IGFBP-7 or IGFBP-7/IGF-1 ratio values and lower IGF-1 levels showed a graded correlation from controls to LVDD to HFpEF. The ROC analysis showed a sufficiently well performance for IGF1, IGFBP7 and the IGFBP7/IGF-1 ratio. In addition, higher IGFBP-1/GF-1 ratios were associated with established markers of diastolic dysfunction including LAVi and the E/E’ ratio. Particularly, an increased LAVi without concomitant mitral valve disease reflects a chronic remodeling process compatible with HFpEF [15]. The lack of correlation to the LVMi and GLS.

Furthermore, we found that elevated IGFBP-7/IGF-1 ratios were associated with elevated NT-proBNP levels, a well-recognized prognostic marker and indicator of elevated ventricular filling pressures among patients regardless of EF [16, 17].

Low levels of IGF-1 have been reported in patients with HFrEF [6]. To our knowledge, the present study is the first to show a significant inverse relationship between IGF-1 serum concentration and the presence and severity of LVDD among patients with preserved EF. Because physiologic effects of IGF-1 suggest potential beneficial effects on cardiac metabolism, cell growth, and cardiac function, GH therapy has already been tested in HFrEF patients with mixed results [18]. These inconsistent findings may be due to a discrepancy between circulating levels of IGF-1 and the activity of IGF-1, related to the complex regulation of IGF-1 activity in vivo where it is bound to IGFBPs. Thus, we hypothesized the ratio of IGFBPs/IGF-1 may serve as a surrogate to better estimate the activity of IGF-1 in patients. Hence, this ratio was tested in the current study.

A recent analysis identified a potential link between IGFBP-7 and hepatic fibrosis [19]. Given fibrosis is an important pathophysiologic mechanism inherent to many HFpEF patients [20], it can be hypothesized that elevation of the IGFBP-7/IGF-1 axis may partly be linked to the increase of cardiac collagen content among patients with HFpEF. This is further supported by correlation to serum sST2 levels, a known marker of increased collagen synthesis by cardiac fibroblasts [20]. Circulating sST2 concentrations are believed to reflect cardiovascular stress and fibrosis, and the biomarker has recently been cleared by the US Food and Drug Administration for use in assessing prognosis in HF [21]. A sST2 level > 35 carries an increased risk of adverse outcomes [22]. In the present study, the IGFBP-7/IGF-1 ratio was significantly elevated in patients with sST2 values above > 35 ng/mL, compared to those < 35 ng/mL.

Serum IGFBP-7 levels are associated with insulin resistance and the risk of metabolic syndrome [23]. In the current study, the IGFBP-7/IGF-1 ratio was significantly increased in patients with metabolic syndrome and/or diabetes compared to those without metabolic syndrome. It has previously been shown that low concentrations of IGF-1 in the circulation increased the risk for developing type 2 diabetes considerably during a 4.5-year follow-up in 615 participants [24]. Compared to other IGFBPs, the affinity of IGFBP-7 to insulin is 500-fold higher [25]. This suggests IGFBP-7 could compete with insulin receptors for insulin binding and interfere with the physiological response to insulin, contributing to insulin resistance and subsequently to development of diabetes and cardiovascular disease [26]. Indeed, diabetes mellitus and metabolic syndrome are known to be associated asymptomatic LVDD as well as HFpEF [27–29].

HFpEF is a clinical syndrome strongly associated with metabolic abnormalities leading to cardiac dysfunction, skeletal muscle deconditioning, and cachexia [30]. The present data support the IGF axis as a promising “cardiometabolic biomarker,” linking cardiac structure and function to metabolic abnormalities in patients with HFpEF. These emerging new biomarkers may complement established biomarkers such as natriuretic peptides, troponins, and sST2, reflecting different pathophysiological pathways. For a patient population presently without evidence-based treatment options, a comprehensive multi-marker approach has the potential to improve patient stratification, prognostication, and guide selection/titration of investigational therapeutics [31].

Several limitations of this study must be acknowledged. The retrospective observational nature of the present study prohibits definitive determination of cause and effect relationships. Second, the present study was a single-center experience with a relatively small number of subjects. Third, longitudinal follow-up data were not available to test associations between the IGFBP-7/IGF-1 axis and clinical outcomes. Moreover, we enrolled consecutive patients referred for elective coronary angiography and echocardiography which may not represent a general population cohort without evidence or suspicious for cardiovascular diseases. In addition, in the control group, NT-proBNP levels were higher than previously reported in healthy controls and comparable levels that have been described in patients with AHA/ACC stage B HF [32], indicating that some degree cardiac dysfunction in this cohort. However, the NT-proBNP levels were still within the normal limits.

Lastly, other IGFBPs can also influence IGF-1 activity and thus the IGFBP-7/IGF-1 axis may not be a complete measure of IGF-1 activity.

## Conclusion

In conclusion, this exploratory, hypothesis-generating study suggests the IGFBP-7/IGF-1 axis correlates with metrics of diastolic dysfunction and may act as a novel biomarker in patients with HFpEF. A rise in IGFBP-7 or IGFBP-7/IGF-1 ratio may reflect worsening diastolic function and adverse cardiac remodeling. Further prospective studies are needed to determine the diagnostic and prognostic value of the IGF axis in HFpEF and the potential role as a therapeutic target.

## Abbreviations

ATP-III: Adult Treatment Panel III
BMI: Body mass index
E: Early diastolic inflow velocity
E´: Early diastolic relaxation velocity
EF: Ejection Fraction
ELISA: Enzyme-linked immunosorbent assays
GH: Growth hormone
HF: Heart Failure
Hs-CRP: High sensitivity c-reactive protein
HOMA: Homeostasis model assessment of insulin resistance index
HFpEF: Heart Failure with preserved ejection fraction
HFrEF: Heart Failure with reduced ejection fraction
IGFBP: Insulin-like growth factor binding protein
IGFBP1: Insulin-like growth factor binding protein-1
IGFBP7: Insulin-like growth factor binding protein-7
LAVi: Left ventricular volume index
LV: Left ventricular
LVDD: Left ventricular diastolic dysfunction
NTpro-BNP: N-terminal pro-B-type natriuretic peptide
oGTT: Oral glucose tolerance test
ST2: Soluble suppression of tumorigenicity-2
